# Realist process evaluation of a co-designed intervention to address the unmet information needs of people with dementia and their family carers

**DOI:** 10.1371/journal.pone.0348633

**Published:** 2026-06-24

**Authors:** Chiara De Poli

**Affiliations:** Department of Social Policy and Care Policy and Evaluation Centre, London School of Economics and Political Science, London, United Kingdom; Taipei Medical University, TAIWAN

## Abstract

Information needs among people with dementia and their family carers remain often unmet, despite policy emphasis on information as the key enabler of choice, agency, and empowerment. An intervention to address these unmet information needs was co-designed and implemented in the North-East of England. This study is a realist process evaluation of the intervention’s implementation through the local community mental health services for older people. The realist process evaluation drew on three strands of qualitative data: longitudinal interviews with the service manager of the implementing services, longitudinal group interviews with practitioners, and a focus group with the co-design working group. Thematic analysis used a deductive approach to identify implementation outcomes (*Reach, Dose, Fidelity & Adaptation, Acceptability, Adoption, Appropriateness, Penetration, Feasibility, Sustainability*), followed by a retroductive approach to interpret data through the realist framework of Context, Mechanisms, and Outcomes. Participants showed different strategies for offering the leaflet in clinical practice (*Reach*), but all appeared to offer it multiple times and at every opportunity (*Dose*). Some adaptation in implementation was identified. The intervention was successfully integrated into practice (*Penetration*, *Feasibility*) and well received by implementers (*Acceptability*, *Adoption*). The intervention was perceived as addressing a genuine need and as compatible with the implementation setting (*Appropriateness*), also in the longer term (*Sustainability*). Nine levels of contextual factors and eight mechanisms appeared to contribute to the observed outcomes. The study offers an in-depth understanding of how information-giving occurs at the level of the individual practitioner and identifies some variation in information-giving practice. Some participants’ approach to information-giving was consistent with a person-centred, relational, and situated approach to care, while others embodied a rational approach to information which did not account for the circumstances of the information recipient. Policy and practice should acknowledge and address this variation to ensure that critical stances on information-giving are widely adopted.

## Introduction

Successive reforms of the welfare system in England have introduced quasi-market mechanisms and consumerist approaches to care [1], positioning information as a key enabler of user choice, agency, and empowerment [2]. This rests on the assumption that if individuals are provided with accessible, high-quality information, typically through formal encounters with professionals or via formal sources such as institutional websites, they will engage with it and act accordingly. Information is therefore expected to support individuals in, for example, make informed choices about care options available, or managing their own health (or the health of the person they care for). Ultimately, this may help people to feel more in control, empowered, confident, and satisfied with the care they receive, and potentially contribute to better outcomes [3,4].

This rational approach to health information, a shorthand for health and care information, which has dominated family care and dementia care policy for about 15 years (e.g., in subsequent national dementia strategies [5–8], in national carers strategies [9–12], in the NHS Long Term Plan [13]), seems to have failed to deliver on its promises. Evidence shows that among people living with dementia and their family carers, needs for information are widespread and often unmet [14–18], leading to negative consequences such as poorer physical and mental health outcomes, avoidable hospital admissions, premature use of residential care, and carer burden [4,18–22].

The health information debate has presented five arguments showing that the rational approach to information is unsatisfactory in the context of family and dementia care. First, the rational approach assumes that written and oral information travels predominantly from professionals to patients and users on official, formal information channels (such as a clinical encounter or a care needs assessment) [23,24]. It does not acknowledge that, in a digital age, information is accessed and shared also online and often in multiple directions, including through peer-to-peer networks and informal routes [25].

Second, information *provision* is considered the key information-related activity that takes place between information providers and users [26]. However, the range of information-related activities is broader than information provision only: it encompasses the activities in which a person may engage when identifying their own information needs, searching for, accessing, and using (or deciding not to use) information [27], as captured by the concepts of information behaviour and ‘information work’ (i.e., the labour of ‘sifting through, interpreting, and dealing with the implications of the information one finds’) [26,28].

Third, a rational approach to information tends to support a system where information is provided in a standardised and generic way and does not allow for tailoring to individual preferences, characteristics, circumstances, needs, and interests [15,29–37]. For example, age may increase the need to access multiple health and care services, which may require navigating different sources of information, but age may also be associated with diminished information-seeking skills. Hence, standardised, one-way information provision strategies may fail to support the least healthy, older people, and those with declining or fluctuating cognitive functions [38,39].

Fourth, the rational approach to information assumes that decisions and actions are by-products of the provision of information, and that responsibility for action (or inaction) lies with the individual, regardless of their characteristics and circumstances. In this context, information seems to replace, rather than support, care [2,40] and the mechanisms (material, cognitive, and psychological) that may trigger decisions and actions are overlooked.

Last, by reducing information-related activities as transactional, mechanistic, and value-free exchanges of generic information, the rational paradigm fails to acknowledge that such activities are intrinsically relational, often emotionally charged, contextually embedded, and require (and produce) situated knowledge [2,40,41].

This work aims to contribute to the debate in favour of a critical approach to health information centred on the concept of information behaviour and one that moves away from the rational health information paradigm.

The empirical component of the study builds on a co-creation initiative in dementia care in the North-East of England (UK) that led to the co-design of an intervention to address the unmet information needs of people living with dementia and their family carers in the study site [42]. The intervention centred on an information leaflet disseminated via a three-armed implementation strategy (local Community Mental Health Services for Older People, primary care, and online).

This article presents the results of the process evaluation of the intervention that was implemented by the local Community Mental Health Services for Older People (CMHSOP). Methodologically, the process evaluation adopts a realist approach [43]: it aims to understand the configurations of mechanisms and contextual factors that shaped the implementation process and brought about the observed implementation outcomes. By taking this approach, this work provides a detailed account of information-giving in action and explores how professionals support the information behaviour of people with dementia and family carers.

### Co-creation in dementia care

I was member of the team involved in a co-creation initiative in dementia care that took place between 2015 and 2021 in the North-East of England (UK). The co-creation initiative involved a constellation of local stakeholders, including people living with dementia and their family carers, health and social care practitioners, service managers, commissioners alongside a research team, encompassing three university-based researchers (including myself) and a local research facilitator.

Grounded in the principles of action research, co-creation activities were organised in three phases [44]. The diagnostic phase (phase 1, 2015–2018) mapped the local dementia care system and identified its strengths and weaknesses. The improvement phase (phase 2, 2017–2018) articulated and designed three interventions to address the priority issues identified in the diagnostic phase. Each improvement area was addressed via a sub-cycle of action research. Local stakeholders with an investment in the respective improvement areas were invited to work with the research team to identify different available options to address those areas, select a course of action, design an intervention, and define its implementation. The planned evaluation (phase 3, 2020–2021) focused on both the improvement interventions and the co-creation initiative. The evaluation of each intervention was designed with the aim of assessing both the outcomes of the intervention and the implementation strategy (process evaluation).

### The intervention

The diagnostic phase of the study identified information needs as one of the most common and frequently unmet [45]. During the improvement phase, local stakeholders agreed to address these unmet needs by designing an intervention to support the information behaviour of people with dementia and their family carers. To guide this work, a local working group was established, comprising commissioners, statutory service providers, third sector organisations providing support and care to people with dementia and family carers, and a professional designer, with the research team providing methodological input. Efforts to involve family carers and people living with dementia through local third-sector organisations resulted in only one family carer joining the group.

The co-design process produced three leaflet prototypes, which were shared and discussed in a consultation exercise with around 20 people with dementia and family carers, planned to address their limited representation on the working group. Feedback from this consultation, together with guidance on best practice for Patient Information Leaflets [22], informed iterative refinements to the preferred prototype. Following local discussion, it was decided to produce a single leaflet for both family carers and people living with dementia presenting a list of local services clustered around four main themes (“I want to speak to someone for advice and support”, “I want to live safely and with the right support”, “I want to know what to do in a crisis”, “Where can I go for more information?”).

For each service, it was agreed to provide only the name and contact details of the organisation provider in keeping with the aim of the leaflet to be a signposting tool, to help people locate possible sources of support. The leaflet was intended to be printed on a two-sided A4 and was designed using a high contrast colour combination.

As part of the co-design process, local stakeholders discussed also how the leaflet would be disseminated in practice. The Community Mental Health Services for Older People (CMHSOP) within the local mental health trust were identified as the first dissemination route because of their central role in diagnosing dementia and providing post-diagnostic support and care planning. At the start of the process, CMHSOP comprised four locality teams, with staff numbers ranging from 26 to 48 (including 5–9 CPNs), and caseloads between 787 and 1,377 patients.

Discussions around the adoption of the leaflet within CMHSOP and its use in clinical practice (defined here as the implementation strategy that enabled its dissemination) were largely dominated by the CMHSOP members of the leaflet working group (i.e., service manager, team managers, and representatives of community psychiatric nurses (CPNs)), not least because the intervention directly affected their services. It was agreed that the leaflet would be made available for CPNs and other team members to use with patients undergoing diagnosis or diagnosed with any form of dementia and/or their family carers. CMHSOP staff were clear that its adoption should *not* be mandatory; accordingly, it was not embedded in the local clinical pathway but was left entirely to professional clinical judgement.

**Fig 1:**
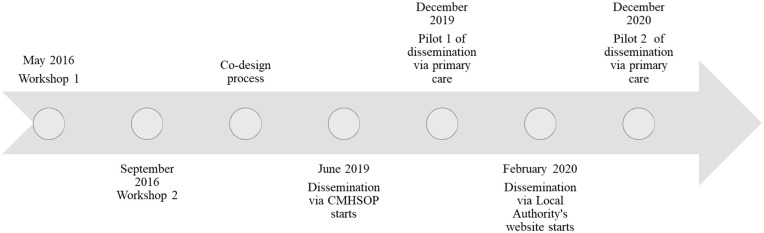
Timeline of the co-design process.

When concerns were raised within the working group that patients were discharged by CMHSOP within 12 months of diagnosis, complementary dissemination routes were identified and implemented by other local organisations (i.e., via primary care, and via the website of the local authority) (Fig 1).

This article reports the findings of the process evaluation of the intervention based within CMHSOP. The intervention comprises both the leaflet and the set of activities that enabled the dissemination of the leaflet among CMHSOP patients (i.e., the implementation strategy).

A protocol paper describes the co-design process that underpinned the intervention [42].

## Methods

### Realist evaluation

The process evaluation was conducted using a realist approach. Realist evaluation posits that interventions work (have successful outcomes) in so far as they introduce appropriate ideas and opportunities (mechanisms) to individuals and groups in the appropriate social and cultural conditions (context), as condensed in the formula [46].


$$\begin{array}{c} \text{Context }(\text{C})\;+\text{ Mechanism }(\text{M})\;\to \text{ Outcome }(\text{O}) \end{array}$$

According to the realist framework, mechanisms are the underlying processes or hidden causal levers of intervention activities that make them work [47]. Power, resources, and ideas can shape the reasoning and responses of intervention participants, which together constitute realist mechanisms [48,49]. Context refers to the conditions likely to enable or constrain the activation of intervention mechanisms. Outcomes are the anticipated and unanticipated consequences that are brought about by the interaction of different intervention mechanisms in different contexts. In accordance with the Medical Research Council (MRC) process evaluation guidance, *Reach*, *Dose*, *Fidelity & Adaptation*, *Acceptability*, *Adoption*, *Appropriateness*, *Feasibility*, *Penetration*, *Sustainability* were the implementation outcomes of interest of the study [50–53].

### Data collection

The study was reviewed and received approval by the HRA West Midlands – South Birmingham Research Ethics Committee (REC reference 16/WM/0397).

The CMHSOP service manager was interviewed twice (online) in July 2019, to understand how the service prepared for the adoption of the leaflet in practice, and again in January 2020, to gather their views about how the implementation was progressing across the locality teams about six months from the roll-out of the intervention.

Participants based within CMHSOP were recruited between December 2019 and June 2021. All participants received an information sheet about the study before taking part. Two rounds of group interviews with clinical staff based at each of the four locality teams within the CMHSOP were then conducted. The first round was held in person in January-February 2020. The second took place online (via Teams) between April and June 2021, due to COVID restrictions. The interviews were followed by an online focus group (via Teams) with the leaflet working group involved in the intervention co-design process (June 2021). Participants in the first round of interviews consented in person and in writing. Participants in the 2021 interviews and focus groups consented remotely, in writing or verbally, if they were unable to sign and return an electronic consent form.

Participants were invited by email to take part in the relevant research activity. Members of the locality teams were recruited via the locality managers and were invited to take part in an interview to discuss how they had used the leaflet in their clinical practice. In the first round of interviews (January-February 2020), 24 practitioners took part across the four locality teams, including five locality managers, 12 Community Psychiatric Nurses (CPNs), three advance nurse practitioners, two community mental health nurses, and one care home liaison nurse. For one participant the role was not provided (Table 1).

**Table 1:** Participants interviewed in January-February 2020.

Locality	Participant Id.	Role
Locality 1	1	Team manager
	2	Advanced nurse practitioner
	3	CPN
	4	Community mental health nurse
Locality 2	1	CPN
	2	CPN
	3	Community mental health nurse
	4	CPN
	5	Team manager
	6	Team manager
	7	Advanced nurse practitioner
	8	CPN
	9	NA
Locality 3	1	Team manager
	2	CPN
	3	CPN
	4	CPN
	5	CPN
	6	CPN
Locality 4	1	Team manager
	2	Advanced nurse practitioner
	3	Care home liaison nurse
	4	CPN
	5	CPN

In the second round of group interviews, one locality team did not reply when invited to take part. Across the other three localities, 22 professionals took part in total, including three locality managers, 12 CPNs, one advance nurse practitioner, one occupational therapist (OT) lead and two OTs (Table 2). For three participants, the role was not provided. Nearly the same set of participants from each locality took part in both interviews. The only exception was one locality where all participants (including the locality manager, newly appointed) were different between the first and the second interview.

**Table 2:** Participants interviewed in April-June 2021.

Locality	Participant Id.	Role
Locality 1	1	CPN
	2	CPN
	3	Team manager
	4	CPN
Locality 3	1	Team manager
	2	CPN
	3	CPN
	4	CPN
	5	CPN
	6	NA
	7	NA
	8	NA
Locality 4	1	Team manager
	2	Advanced nurse practitioner
	3	CPN
	4	CPN
	5	CPN
	6	CPN
	7	CPN
	8	Clinical lead OT
	9	OT
	10	OT

The focus group included 20 participants, one was a family carers and the rest were professionals and practitioners who were involved in the leaflet working group and/or in the implementation of the leaflet via the CMHSOP (Table 3). The focus group was an opportunity for the CMHSOP to discuss how the leaflet was adopted in practice and for the working group to reflect on the co-design experience. Two representatives of local third sector organisations, one hospice nurse, and two practice managers were invited but did not attend.

**Table 3:** Focus group participants.

Organisation	Role	N. of participants
Lay participant	Family carer	1
Local Authority	Commissioning Policy & Planning Officer, Adult & Health Services	1
NHS trust	CMHSOP locality managers	3
NHS trust	CPNs	9
NHS trust	CMHSOP service manager	1
Primary care	Assistant practice manager	1
Third sector	Local Services Manager	1
Third sector	Local Services Manager	1
Third sector	Mental Health Carer Support and Development Worker	1
Fire services	Community Safety Team Leader	1

### Data analysis

The interviews and the focus group were recorded with consent from participants, professionally transcribed, and suitably anonymised. The transcriptions were then analysed thematically using NVivo12. An initial two-level codebook was developed deductively with codes derived from the MRC process evaluation guidance [53]. The three parent codes were defined as follows

- Outcomes of the implementation strategy, i.e., process or quality measures to assess the impact of the implementation strategy
- Mechanisms underpinning the implementation strategy, i.e., what made the implementation possible
- Context, i.e., anything external the intervention aﬀecting its implementation

Each parent code was further organised in child codes derived from relevant methodological guidance for process evaluations [50–54] and realist evaluations [55–57]. As the analysis progressed, the operationalisation and the analytical scope of the sub-codes were gradually refined to help adjudicate between competing codes.

The next phase of the analysis was guided by a retroductive approach that entailed moving backwards and deeper from events which were captured in the empirical data to the hidden and underlying mechanisms and contextual factors that could help explain the observed events [58]. The realist CMO configuration (Context + Mechanism → Outcome) was used as a heuristic to systematise the interpretation process [59]. The final codebook is provided in Table 4.

**Table 4:** Overview of codebook.

Code	Sub-code	Definition
1. Outcomes of the implementation strategy [51,52]	• Reach	Whether the intended population group is targeted by the implementation strategy and how
	• Dose	Quantity of the intervention that was implemented
	• Fidelity & Adaptation	Whether the intervention was delivered as intended
	• Acceptability of the intervention by implementers	Perception among implementers that the intervention is agreeable, palatable, or satisfactory
	• Adoption of the intervention by implementers	Implementer’s intention, initial decision, or action to implement the intervention
	• Appropriateness of the intervention as perceived by implementers	Perceived fit, relevance, or compatibility of the intervention with the implementing setting Perceived fit of the intervention to address a particular issue
	• Penetration of the implementation strategy among implementers	Integration of intervention with the implementing setting
	• Sustainability of the implementation strategy	Extent to which the intervention is maintained or institutionalized within the implementing setting
	• Feasibility of the implementation strategy	Extent to which the intervention can be successfully used or carried out within the implementing setting
2. Context [50,54–56]	• Outer context	Wider social, cultural, political, policy context in which the intervention is implemented
	• Inner context	Characteristics and culture of the implementing organisation
3. Mechanisms [57]	• Resources	Component of the intervention which is introduced in a context
	• Reasoning	Human volition, ideas, behaviours

Results are reported in accordance with the relevant items of the Standards for Reporting Implementation Studies (StaRI) checklist [50,54] (see S1 File. Standards for Reporting Implementation Studies (StaRI) checklist).Direct quotations reported in the Results are marked with the following notation: *T…* for the time of data collection (T1 for data collected in the first round between January and February 2020; T2 for data collected in the second round between April and June 2021); *L…* for the locality where the data were collected; *P…* for the participant identification number.

## Results

### The implementation process

Participants described two main ways of using the leaflet in practice (*Reach*). Some offered it to all patients and family carers

*“We give it to everyone. And during the highest point of the pandemic, when people were not getting face to face (appointment), then we’d send it out in the post.” [T2, L1, P1]*

Others were more selective and highlighted situations in which they would *not* hand out the leaflet, at least initially

*“Sometimes I don’t give that out on their initial assessment because it talks straight away about dementia, and we’ve got people who have come to us with memory problems that might not go on to be given that diagnosis. So, it depends on the person and your assessment of that person, whether it’s relevant at that point or whether it’s something that needs to go out at a later stage.” [T1, L3, P4]*

Similarly, they would not offer the leaflet directly to patients who had not accepted their diagnosis. In those instances, they would offer it to the family instead

*“If someone was adamant, ‘*There’s nothing wrong with me…’*, I would probably look to the family,* ‘Look, I’m going to give you this information. It might be that you want to keep a hold of it’*, rather than giving it to that person who’s then going to have that reminder, and they’re adamant there’s nothing wrong with them.” [T1, L3, P4]*

In terms of *Dose*, participants highlighted that they would offer it at every suitable opportunity

*“What I’m finding is, you give it an initial assessment but then some people, because of the nature of their illness, they misplace it, or they forget that they even got it. That’s why we give it out a few times, and just to make sure they definitely have got the numbers (on the leaflet).” [T2, L1, P1]*

In their view, offering the leaflet multiple times was indeed good practice

*“We drum it in several times (…). So, repetition sometimes is a good thing.” [T2, L3, P5]*

Participants made two sets of comments relevant to the *Fidelity and Adaptation* criteria. Firstly, some commented that the leaflet was sometimes printed in black and white, rather than in colour, which was the design option identified to maximise accessibility. Over time, in practice, the colour of the leaflet became its distinguishing feature, making it easily recognisable to patients and professionals alike. When the leaflet was not available in colour, its recognisability was hindered

*“Some of our admin are obviously printing them out in black and white, and they shouldn’t be, because the whole idea of being a different colour was so it stood out from these bits and pieces.” [T1, L1, P1]*

The second set of comments related to how professionals used a contact details box on the front cover of the leaflet. In practice, there was a considerable degree of variation in the way in which professionals would complete this box. This approach appeared to deviate from the intended implementation strategy, yet it was acknowledged that this level of adaptation was consistent with an individualised, person-centred approach to care

*“I’m quite reassured that there isn’t any consistency of whether or not there’s a named person or ‘the duty worker’ on that front, and the reason for that is, it depends where and how long somebody’s been known to our services. So, if someone’s got a CPN, has got a really good relationship with them, I would expect that name to be on there. But if someone’s been seen by, maybe, a psychiatrist, a consultant, a doctor, and they (…) can be a point of contact and (…) put that on the front. So, the joy of that front bit is to have that flexibility...” [Focus group, June2021, Service manager]*

### Outcomes of the implementation strategy

Rates of *Adoption* of the leaflet within CMHOPS staff were high

*“The organic nurses (i.e., nurses working in the organic cells of the team) have been using it. The medics have and psychologist has. Don’t think our ANPs (Advanced Nurse Practitioners) will have used it so much because we tend to do the post-diagnostic and the follow-ups and the reviews as nurses.” [T1, L2, P4]*

The introduction of the leaflet in practice was well-received by participants (*Acceptability*)

*“As a (NHS Foundation) Trust, we’re required to give out quite a lot of information (…) and sometimes that drives us round the bend, in terms of how much information we have to give out. We’ve got to be really careful and selective about when we give it, and there is pushback from our staff and our nurses about the need to do that as part of that pathway, which we have to follow. There’s been no pushback on this one.” [Focus group, June 2021, Service manager]*

Participants felt that the leaflet could improve how the service supported the information behaviour of patients and families and address their need for information, and this may contribute to explaining the high rate of uptake and good acceptability

*“We’ve been involved in looking at what would be valuable for our patients. (…) We know in terms of what we offer as a mental health service, it’s sometimes limited (…). I see that we don’t always keep up to date. So, it’s great to have something that we can work with the patients and steer them towards.” [Interview, February 2020, Service manager]*

CMHSOP staff seemed to adopt the leaflet because it had a good fit and was relevant for their practice, since it could support their information-giving and it was consistent with their skill set, roles, and responsibilities. They also felt that the leaflet could be useful and appropriate for the patients. In terms of content, participants pointed out how the leaflet was simple and accessible, which was crucial given the target population

*“It’s not too bulky. There’s just the right amount of information. It’s not jargonized.”[T1, L2, P4]*

They also highlighted that the key feature of the leaflet that made it particularly fit for purpose was the right balance between being short and yet comprehensive

*“The fact that it is one page is a huge selling point, because it’s one point of reference (…) with all of those helpful numbers on.” [T1, L2, P6]*

Participants agreed with the deliberate decision made during the co-design process to avoid using the word dementia in the title of the leaflet, which could have put off at least some people

*P4: “I think even when they’ve got a diagnosis… just saying memory is better than having the word dementia on.”*

*P1: “It does say who to contact if you feel you need help with your memory, or if you or a loved one have a diagnosis of dementia. So, it doesn’t specifically say that the person has dementia, which is good, because some people don’t like that” [T1, L1]*

Equally, professionals highlighted that some information included in the leaflet was already covered at least partially by other leaflets and materials included in information packs they were handing out. Some patients may have found this confusing and difficult to navigate, but others may have benefited from finding the same information in different leaflets

*“You find yourself just revisiting parts of it, depending on what the clinical picture is. It’s not always the case of streamlining so that people don’t get duplication. I think sometimes it just reinforces things.” [T1, L3, P3]*

They also highlighted two limitations of the leaflet. One was its lack of recognition of diversity

*“There’s no advice and support for the LGBTQ+ community (…). There are more LGBTQ+ people coming through the service now.” [T2, L4, P1]*

The other limitation related to the geographical coverage of the services listed in the leaflet. For patients living on the borders of the geographical area covered by the service, or for care home residents who had moved to the area from another local authority, some of the information in the leaflet was less relevant.

The adoption of the leaflet in the work and practice of the CMHSOP teams was not only perceived as appropriate by participants, but also as feasible (*Feasibility*)

*“It’s just a really useful tool and we’ll continue to use it. As long as it gets updated, we’ll continue to send out the updates. It’s just become part of our work and practice now.” [T2, L1, P3]*

One of the reasons for the successful adoption of the leaflet was that it was made available, but its use was not strictly prescribed (as also evidence by the *Dose* criteria). The explicit choice was to allow professionals to exercise their clinical judgement about when and how to hand it out

*“Everybody develops a way of giving the information, and it depends on the situation, the people that you’re sitting in front of, and how it progresses.” [T1, L3, P3]*

Participants described different strategies they used to offer the leaflet. In some cases, they highlighted specific sections they felt were particularly relevant to the patient

*“If you think somebody might be at risk of wandering out, then you would say, “*Oh, this is really worth looking into*” to highlight the Herbert Protocol rather than just give it to them…” [T1, L1, P3]*

When they felt it was necessary, they provided a fuller overview

*“The lady I’ve just had in the clinic, I’ve gone right through it with her. She* needed *to have a go right through… And the lady I had in before, I just gave it to her at the door and said,* ‘It’s a night time reading for you’*.” [T1, L2, P6]*

Professionals also used the leaflet to help people identify their information needs and to raise awareness of local care and support services

*“Problem is, sometimes carers don’t know what they need. This (the leaflet) gives them an explanation of what services do. (…) And I always say to them ‘*You’ve got your way to get where you need to go. Have a look at this because it tells you what different people offer. That might just help you to find the right support’*.” [T1, L2, P4]*

The *Penetration* of the adoption of the leaflet in practice was facilitated organisationally in two ways. First, the leaflet fitted in with the CMHSOP policy of offering information packs to each patient on the caseload, at various points along the clinical pathway. The leaflet was then added to these packs since it would have provided valuable information

*“The leaflets go in the packs as well. We have packs that we give out to people, so you get a pack at initial assessment, then you get another one at diagnosis as well. So they’re in the packs…” [T2, L1, P3]*

Second, participants described how the introduction of the leaflet was cascaded from the service manager to the locality managers and then to the locality teams*.* The locality managers actively promoted the leaflet across members of staff, including new ones, and locality teams were regularly reminded about the availability of the leaflet

*“We shared it in our community meetings. We shared it via email. And went in all of the leaflet racks. (…) We brought it home really and just shared it and banged the drum a little bit, because sometimes we need to tell people things more than once in team meetings.” [W1, L2, P6]*

The *Sustainability* of the implementation strategy had an organisational and a financial dimension that were *designed in* during the co-design process. Participants were aware of previous directories of services which had not been maintained over time, resulting in outdated and inaccurate information. This underscored the importance of designing for sustainability (*Sustainability*)

*“Past experience from these big directories was, they were constantly going out of date. They were never up to date, and people were ringing numbers or contacting people, and they didn’t exist. I think that was one of the challenges for yourselves (the research team)… coming up with something that was relatively easy to use, but that wasn’t simply going to go out of date within months of being produced.” [T1, L1, P1]*

The local Dementia Strategy Implementation Group, led from the local authority, not only contributed to the development of the leaflet, but also agreed to take responsibility for its regular update and for notifying relevant local stakeholders when an update was released, including the CMHSOP manager

*“We have a dementia strategy implementation group, and the leaflet will come (to the group) on a regular basis. We meet on a quarterly basis, so there’ll be a standard agenda item, and we will update it as and when we feel it’s necessary. (…) Any changes that are being made, the leaflet itself will be given a new version number and a new date. Hopefully, that won’t be very often because the numbers will be the same all the time.” [Focus group, June 2021, Commissioner]*

From a financial perspective, the intervention was considered low cost. Copies of the leaflet were printed out by the administrative staff who were usually in charge of preparing the information packs. Excluding the peak of the COVID pandemic, leaflets were handed out in person, and no postal costs were incurred.

### Mechanisms in context

**Fig 2:**
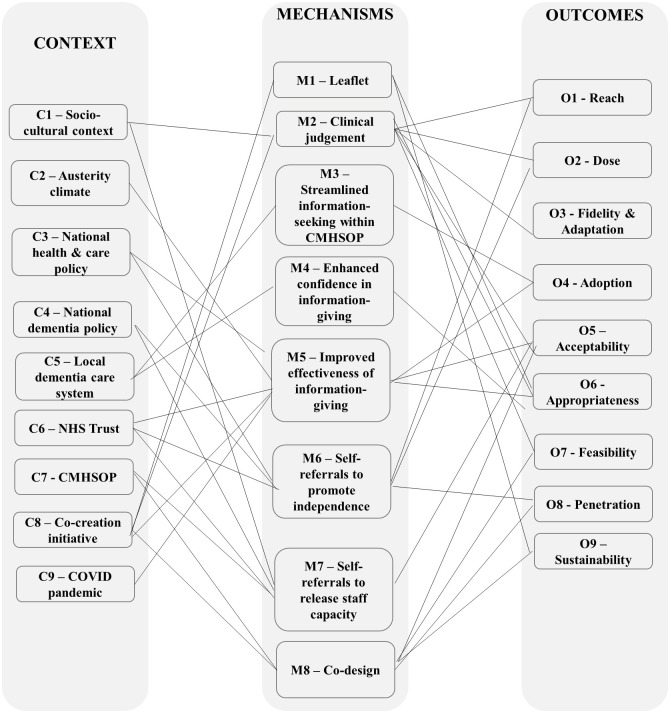
Overview of the Context-Mechanism-Process configurations.

Nine layers of contextual factors shaped the implementation of the intervention (Fig 2).

The intervention interacted with the wider social and cultural context (C1) in which it was implemented. It aimed to support information-giving by professionals involved in dementia care who were aware of public perceptions about and, sometimes, stigma associated with dementia and mental health services in general

*“I’ve had people who would only see someone if it says memory team on it, not mental health.” [T1, L3, P5]*

The intervention was implemented in the context of a decade of financial austerity, which affected health and social care provision (C2). In 2019, the local clinical pathway was revised to reduce the length of time a patient was monitored by the CMHSOP, resulting in a halving of each professional’s caseload. The policy change may have had a strong clinical rationale, but organisational pressures on the CMHSOP may also have contributed.

The intervention was designed and implemented in the context of successive reforms of the health and care system in England, promoting user choice, active agency, and empowerment (C3). It was also meant to be in line with the national dementia policy (C4), which promoted, among other things, post-diagnostic care, including “information on what post-diagnosis services are available locally and how these can be accessed” and “access to relevant advice and support to help and advise on what happens after a diagnosis and the support available through the journey” [6].

The intervention was introduced in a complex and fragmented local dementia care system (C5), involving different sectors (health care, social care, third sector) and different organisations within the same sector (e.g., primary care, community care, mental health). It was intended to help people understand and navigate the system, to smooth out transitions (e.g., when patients were discharged from CMHOP to the care of their GP), and to improve service integration (e.g., between CMHSOP and social care).

The intervention was adopted by the CMHSOP in an NHS Trust (C6) that had a Trust-wide dementia care pathway in place since 2009. The pathway was prescriptive about what information the patients should receive at different points and, indeed, the information packs were designed to ensure consistency of information provision and compliance with regulatory requirements. In practice, an informal pathway developed over time to work alongside the formal pathway, and the leaflet was added to the latter

*P3: “Things get unofficially added to it over time. You start dishing out leaflets and it will grow exponentially.”*

*P4: “Obviously that needs to be reviewed, because we don’t want to duplicate things. Because we’d want to make it accessible for people, but not overwhelm them with too much information, especially if it’s duplicated in different places.” [T1, L3]*

The locality teams within the CMHSOP constituted a further contextual layer (C7). During data collection it became apparent that the largest locality team had developed and was using a locality-based information leaflet which was not used by the others. When this was brought up with another team, they highlighted that each locality had a distinctive organisational identity and set of resources (e.g., staffing levels) that shaped their ways of working, also in relation to information-giving

*“Although we’re in the one area, we’re a locality (…). And sometimes, the ways of working, although we should all be working together, we often work very differently. (…) Because in numbers we’re smaller, the way that we organise things is tailored really to serve us. (…) Over the years, things have evolved. We’re just a bit different. As different as everybody else is.” [T1, L4, P1]*

The co-creation initiative, which was acknowledged by participants at various points in the data collection process, represented another contextual layer in which the intervention was implemented (C8).

Finally, part of the implementation took place during the COVID pandemic (C9), which had a significant impact on the level of provision and mode of delivery of all health and care services in the study site, including the CMHSOP.

Turning now to the mechanisms underpinning the intervention, the leaflet represented the material *resource* that was introduced by the intervention in the implementation context (M1) and interacted with seven lines of *reasoning* of participants.

The implementation strategy focused on leveraging the clinical judgment of individual professionals (M2) who had the autonomy to determine whether and when to distribute the leaflet, in alignment with their professional roles and norms. This approach was consistent with the person-centred model of care adopted by the service, which emphasised flexibility and adaptation to meet each patient’s unique needs and circumstances

*“It’s about assessing each person on an individual basis and deciding what you’re going to do with that information at that point. (…). It’s about, as a clinician, making that judgment.” [T1, L3, P4]*

The leaflet, as an information resource (M1) used according to clinical judgement (M2), both streamlined professionals’ own information-seeking processes (M3) and enhanced their confidence in the quality of the information they shared with patients (M4)

*“We just do accumulate these numbers over time, but the amount of times that I’ve had to sit and google numbers and then… you know what Google is like, it brings up all kinds of different searches. So, finding the number for our area for whatever service is really difficult, so having it all on that sheet is really helpful. [T2, L1, P4]*

The leaflet was also perceived by professionals as a resource that could make their information-giving practice more effective (M5)

*“A lot of these people have said to you (the researcher, who interviewed people with dementia and family carers living in the area),* ‘We don’t get this information’*. I think they did. They didn’t get it effectively, perhaps, and that’s what the leaflet’s driving at. A lot of the time we get accused of not giving people information, and we’ve had it maybe four or five times already. But that’s fine. We just keep bashing away with it.” [T1, L3, P3]*

The leaflet aligned with the emphasis on user choice, active agency, and empowerment promoted in national dementia strategies and health and social care policies since the 2014 Care Act. At the organizational level, this policy shift was implemented through a new self-referral pathway, enabling patients to access support from third sector organisations without needing a referral from the CMHSOP. On the one hand, participants framed the self-referral pathway as a strategy to promote independence and empower patients (M6)

*“We try and encourage people to self-refer because… But it’s a catch 22. Obviously, you don’t want to take people’s independence and there’s always a need for us to fill stuff in, but, actually, if somebody’s got a carer or a relative or they can do it themselves, we encourage them to do it themselves.” [T1, L2, P6]*

On the other, it was also deployed to help free up staff capacity (M7)

*“I would do a four-page referral to (local service provider), (but) I* can’t *do that for every patient I see. It’s too time consuming, (…) If somebody’s under a lot of stress and would find it difficult to ring up, I’ll do it. I don’t do it for everybody like I used to.” [T1, L2, P4]*

The co-design approach that underpinned the intervention and its implementation strategy constituted the last mechanism that helped explain the implementation outcomes (M8).

## Discussion

This article delves into information-giving practices by health professionals in the context of actual clinical encounters. Research into information-giving has mainly focused on *what* information to provide [60–62] and on the effectiveness of different information-giving strategies (e.g., [63,64]). The in-depth exploration of *how* information-giving at the micro-level of the individual professional unfolds has received minimal attention, in general and in the context of dementia care in particular, and this paper aims to fill this gap.

Empirically, this work draws on a co-designed intervention aiming to support the information behaviour of people with dementia and their family carers living in a local health and care system in the North-East of England. Methodologically, this work is a realist process evaluation that was carried out with the aim of generating evidence about how the intervention was implemented.

This article makes five contributions, the first of which is empirical. It provides a detailed account of how information provision unfolded in practice, from the point of view of the professionals involved, and it shows to whom, when, how, and for what purpose information is provided.

In terms of *whom*, the findings show that every patient would have received the leaflet at some point while on caseload of the service during the implementation period. How this was achieved in practice, though, seemed to vary depending on the individual professionals. Some offered the leaflet to *every* patient at *every* opportunity and, in doing so, adopted a blanket approach to information provision. Other professionals adopted a targeted approach, i.e., they used their clinical judgement to decide to whom and when they would hand out the leaflet. A blanket approach may perform well in term of reach, but it seems problematic because it does not take into account preferences, needs, and circumstances which are known to affect individual readiness and willingness to engage with information [45]. These issues were mitigated by the targeted approach. When professionals adopt this strategy, they are mindful to avoid the information resistance that patients may exhibit when they feel that the information offered is not relevant for them or when their preference is for *not* receiving any information.

In relation to *when*, the leaflet was intended to be handed out opportunistically at any clinical encounter and indeed professionals sought multiple opportunities to support the information behaviour of patients. This feature of the implementation strategy seems important in the context of dementia care, given the progressive nature of the disease and the multiple areas of care needs it affects, which may change over time.

In relation to *how* information was given, professionals showed three different approaches. In some instances, they simply handed out the leaflet, in others they gave a quick overview, leaving the patients or they families to engage with it. In some other cases, they signposted specific elements of the leaflet that they felt the patients and the families would have particularly benefited from (such as the Herbert Protocol if the patient was at high risk of wandering). In deploying these different strategies, professionals seemed to consider, or to make assumptions about, the level of self-efficacy of the patients or their family carers, their psychological and cognitive status, and their personal and family circumstances. Those in (or perceived to be in) a position of relative disadvantage, e.g., because of their particular circumstances (e.g., someone living with dementia without family support, advanced cognitive impairment, or overburdened carers) were more actively supported by professionals than others who seemed to show high levels of self-efficacy, were at an early stage of the condition, or could rely on a support network. Professionals seemed to use the same set of criteria to adjudicate whether their information-giving should be followed up by a professional referral or whether they could leave the patients to initiate a self-referral as and when they felt it was needed. Although the latter was seen as a suitable approach to maintain the person’s independence and preserve their agency, it would be simplistic to assume that every patient, however high their level of self-efficacy or their favourable circumstances, would contact every relevant service. While material, emotional, relational, and cognitive resources can provide significant advantages, they may not enable people to navigate multiple referral processes [65].

These empirical findings tie into the theoretical debate on information provision, which represents the second contribution of this work. When information was given to each patient in a generic way, with a signposting aim, leaving the individual with the responsibility to engage with it and decide on the course of action to take, professionals endorsed a rational approach to information provision. For them the leaflet was an end in itself [66]. This approach – which may be a compound cause and consequence of the national and local policy and organisational contexts in which professionals operated (large caseloads, time pressure, burdensome referral processes) – is not consistent with a person-centred and individualised approach to care. It seemed to serve the information provider rather than the recipient: it required minimal input from the providers (e.g., in terms of time spent with each patient, and effort, as the leaflet effectively was made available to them ready to use), but substantial inputs from the patients, in terms of skill, energy, and resources [64].

Conversely, professionals who were selective about what information to give, to whom, when, and how, demonstrated a practice consistent with a critical approach to information provision, one that involves discussion and deliberation, and that happens in the context of person-centred and relational care [2,40,66]. For them, the leaflet was a material resource on which they could rely to support patients and carers across different phases of their information behaviour [45]. Ultimately, the empowerment of patients and carers that the health and care information agenda seeks to achieve is the outcome of trusting relationships between patients, families, and care professionals, involving exchanges of information over time, not of a leaflet.

The third contribution of this work is to the evaluation field where information leaflets are often used as a standard example of a ‘simple’ intervention [53]. The results of this process evaluation challenge this conceptualisation. The unfolding of the implementation process and the related outcomes can be explained by the interaction of a complex, layered context and multiple inter-related mechanisms that the realist approach helped to pin down. Some mechanisms (e.g., clinical judgment of professionals) triggered expected and desirable outcomes (e.g., dose, fidelity, and adaptation). Other mechanisms were unexpected but still led to positive outcomes: for example, the intervention scored well on the appropriateness criteria also because it helped to increase professionals’ awareness of local provision, which in turn gave them confidence to provide information. This point also highlights how information-giving by professionals requires them to engage in successful information-seeking, and the leaflet supported them in this respect. Some other mechanisms were unexpected and may have led to undesirable consequences, such as the leaflet being used as a shortcut to self-referrals.

Hence, policy and practice should turn their attention to harnessing both contextual factors and mechanisms that, when activated, can lead to desirable outcomes of the implementation of an information intervention, or conversely, to mitigating the factors that can hinder it with unwanted outcomes. The final contribution of this work is, therefore, in terms of changes in policy and practice.

Adequate, long-term funding and staffing of services are the cornerstone of any policy and practice that seriously seeks to address the complexity of information provision. Longer and repeated clinical appointments with the same professional would allow developing and sustaining care relationships which, among other things, would enable information-giving sensitive to the circumstances of the patient and family, and would allow for better responses to information needs as they arise.

In the short term, streamlined referral processes and information-sharing between providers could be considered as a quick fix: they could facilitate the provision of information by professionals working in overstretched services, while supporting the information behaviour of information users. Also, a coordinated and organic information strategy with clear organisational responsibilities between service providers (such as the three-armed implementation strategy developed in this study) could provide multiple opportunities for timely, if not proactive, information provision at virtually no cost.

Finally, this work provides a practical example of how an intervention to support health information can be co-designed and sustained in the current policy and service context. In the study site, the Dementia Strategy Implementation Group—an established inter-institutional body—took ownership of the leaflet, updating it and maintaining institutional awareness among local stakeholders. Within the CMHSOP, the leaflet became a standing item in locality and service meetings, physical copies were stocked in clinics, and new staff were introduced to it during induction. Embedding the leaflet within electronic health records, with automated prompts, could further support sustained use by alerting staff to offer it to new patients and to existing patients who would benefit from receiving the latest version.

This work also highlights options for improving the intervention itself, for example in relation to the information needs of specific groups at risk of disadvantage and care inequalities (such as the LGBTQ+ community). Future revisions of the leaflet could consider how to meet the information needs of such groups, while maintaining its concise and accessible format. A co-design process with the relevant communities would be essential to include tailored content and dissemination strategies via organisations, venues, or channels most likely to reach these groups.

Turning now to strengths of the work, data collection followed a longitudinal design that provided insights into the implementation process as it unfolded, which helped reduce recall bias. The study involved a mix of professionals working in different clinical roles at different levels of seniority in the implementing site. This is a strength of the work because it provided an understanding of the implementation process from both an operational and a strategic perspective. This work adhered to established methodological guidelines in implementation science and evaluation [59,67], with results reported according to the recommended StaRI checklist [50] – another merit of the work.

The study was not without its limitations, however. The data collection was shaped by factors related to the type of study participants (busy professionals with clinical roles in community-based mental health services), staff turnover, and the broader context in which the study was conducted (i.e., the COVID pandemic). As a result, one locality team did not take part in the second data collection phase.

Recruitment was mediated through organisational gatekeeping, with invitations cascaded by service managers, which constrained the researcher’s control over the process and may have introduced an element of selection bias. While many participants were motivated by genuine interest, some engagement may have been performative, reflecting expectations of their organisation and the study. Most interviews were conducted in group settings, which, while reducing participant burden, posed challenges in managing group dynamics and navigating existing hierarchies. Power imbalances were evident, with locality managers more likely than junior staff to voice perspectives on sensitive issues (e.g., staffing levels, resource allocation).

The study was conducted within community-based mental health services in a single locality in the North-East of England, which hosted the co-creation initiative and thus represented the context of the intervention. While the intervention itself may not be directly replicable in other settings, the mechanisms identified through the CMO configurations are potentially transferable. This reflects a key strength of realist evaluation, which focuses on understanding the mechanisms underpinning an intervention rather than treating an intervention as a fixed entity.

Another limitation of this study is the lack of activity data, e.g., number of leaflets distributed or patients reached by the intervention. This was due to the absence of a data infrastructure for recording this. However, the findings of this study suggest that such figures would likely have been affected by double or multiple counting and by naturally occurring variations in coding practices across teams and practitioners, and therefore would have had limited interpretive value.

As a process evaluation, this work does not provide evidence of the outcomes of the intervention. Among people living with dementia and their family carers, the intervention was expected to improve outcomes such as their satisfaction with the available information about local services (e.g., in terms of timing and quality of information), their awareness of local services and, in the longer term, their access and use of services. The intervention was expected also to impact access and use of services listed on the leaflet, measured in terms of volume and appropriateness of referrals (e.g., with respect to the type and level of need). Future work in this field could aim to complement a process evaluation with an outcomes evaluation.

## Conclusion

The discourse on health and care information as the key enabler for patient choice, independence, and empowerment dominates policy, practice, and research in dementia care, and beyond.

This work is an in-depth exploration of information-giving by professionals involved in diagnosing and providing post-diagnostic support for people with dementia and their families. At a first level, it offers a descriptive account of how the implementation of an information intervention unfolded in practice, which is an important contribution in itself: the effects of an intervention can be limited either because of flaws in its design or because of poor implementation [53]. Distinguishing between the two is essential so that mitigating actions can be designed accordingly.

At a deeper analytical level, this work identifies the configurations of contextual factors and mechanisms that underpinned the actual implementation of the intervention. These factors, their contextual contingency, the implementation circumstances, their causal associations, more than the intervention itself, should be given due consideration by anyone interested in the transferability of the intervention.

## Supporting information

S1 File Standards for reporting implementation studies (StaRI) checklist. (DOCX)
