# Supplementary material for: Realist process evaluation of a co-designed intervention to address the unmet information needs of people with dementia and their family carers
Source: PLoS One. 2026 Jun 24;21(6):e0348633. doi: 10.1371/journal.pone.0348633 (PMC13293420; doi:10.1371/journal.pone.0348633)
Supplement: S1 File — (DOCX) [file pone.0348633.s001.docx]

**S1 Supporting Information. Standards for Reporting Implementation Studies (StaRI) checklist**

| **Checklist item** | **Reported on page, in section** |
| --- | --- |
| 1. Title | p. 1 |
| 2. Abstract | p. 2 |
| 3. Introduction | p. 4, Introduction |
| 4. Rationale | p. 6, Introduction |
| 5. Aims and objectives | p. 6, Introduction |
| **Methods: description** | |
| 6. Design and key features of the evaluation | p. 10, Methods - Realist evaluation |
| 7. Context | p. 6, Co-creation in dementia care  p. 7, The intervention |
| 8. Target sites | p. 7, The intervention  see also protocol paper [44] |
| 9. Implementation strategy | p. 7, The intervention |
| 10. Sub-groups | Not applicable to this study |
| **Evaluation** | |
| 11. Outcomes | p. 11, Methods - Data collection |
| 12. Process evaluation objectives and outcomes | p. 11, Methods - Data collection |
| 13. Economic evaluation | Not applicable to this study |
| 14. Sample size | p. 7, The intervention  see also protocol paper [44] |
| 15. Methods of analysis | p.14, Methods - Data analysis |
| 16. Sub-group analyses | Not applicable to this study |
| **Results** | |
| 17. Characteristics | p. 11, Methods - Data collection |
| 18. Outcomes | p. 16, Results - Outcomes of the implementation strategy |
| 19. Process outcomes | p. 25, Mechanisms in context |
| 20. Economic evaluation | Not applicable to this study |
| 21. Sub-group analyses | Not applicable to this study |
| 22. Fidelity/adaptation | p. 17, Results - Outcomes of the implementation strategy  p. 30, Discussion |
| 23. Contextual changes | p. 11, Methods - Data collection  p. 26, Mechanisms in context |
| 24. Harms | Not applicable to this study |
| **Discussion** | |
| 25. Structured discussion | p. 30, Discussion |
| 26. Implications | p. 30, Discussion |
| **General** | |
| 27. Statements | p.37, Declarations |
