# Supplementary material for: Realist process evaluation of a co-designed intervention to address the unmet information needs of people with dementia and their family carers
Source: PLoS One. 2026 Jun 24;21(6):e0348633. doi: 10.1371/journal.pone.0348633 (PMC13293420; doi:10.1371/journal.pone.0348633)
Supplement: Attachment — Submitted filename: Responses to reviewers Dec 2025 FINAL.docx [file pone.0348633.s003.docx]

Chiara De Poli

London School of Economics and Political Science

[c.de-poli@lse.ac.uk](mailto:c.de-poli@lse.ac.uk)

28 November 2025

Dear Editor,

Thank you for the opportunity to revise my manuscript *Realist process evaluation of a co-designed intervention to address the unmet information needs of people with dementia and their family carers.* I have carefully considered your comments, as well as those of the reviewers, and have addressed them in detail in the revised manuscript and in the accompanying response document. I hope that the revisions are satisfactory and address the concerns raised.

Thank you for your time and consideration.

Best regards

Chiara De Poli

**Journal Requirements:** **When submitting your revision, we need you to address these additional requirements.**

1. **Please ensure that your manuscript meets PLOS ONE's style requirements, including those for file naming. The PLOS ONE style templates can be found at**

[**https://journals.plos.org/plosone/s/file?id=wjVg/PLOSOne_formatting_sample_main_body.pdf**](https://journals.plos.org/plosone/s/file?id=wjVg/PLOSOne_formatting_sample_main_body.pdf) **and**

[**https://journals.plos.org/plosone/s/file?id=ba62/PLOSOne_formatting_sample_title_authors_affiliations.pdf**](https://journals.plos.org/plosone/s/file?id=ba62/PLOSOne_formatting_sample_title_authors_affiliations.pdf)

 I have revised the manuscript to ensure it meets PLOS ONE's style requirements.

2. **In the online submission form, you indicated that “The data that support the findings of this study are available from the corresponding author upon reasonable request”.  All PLOS journals now require all data underlying the findings described in their manuscript to be freely available to other researchers, either a. In a public repository, b. Within the manuscript itself, or c. Uploaded as supplementary information. This policy applies to all data except where public deposition would breach compliance with the protocol approved by your research ethics board. If your data cannot be made publicly available for ethical or legal reasons (e.g., public availability would compromise patient privacy), please explain your reasons on resubmission and your exemption request will be escalated for approval.**

The study reports qualitative data collected with in-depth interviews with professionals working in health and social care and was reviewed and approved by one of the Research Ethics Committees of the Health Research Authority. in accordance with the HRA REC ethics approval and to protect participant confidentiality, the data cannot be shared outside the research team.

3. **Please include your full ethics statement in the ‘Methods’ section of your manuscript file. In your statement, please include the full name of the IRB or ethics committee who approved or waived your study, as well as whether or not you obtained informed written or verbal consent. If consent was waived for your study, please include this information in your statement as well.**

I have now included a complete ethics statement in the Methods section of the manuscript. The statement provides the full name of the ethics committee that approved the study, and details of how informed consent was obtained from participants.

4. **Please include captions for your Supporting Information files at the end of your manuscript, and update any in-text citations to match accordingly. Please see our Supporting Information guidelines for more information:** [**http://journals.plos.org/plosone/s/supporting-information**](http://journals.plos.org/plosone/s/supporting-information)**.**

I have included the caption for my Supporting Information files at the end of the manuscript, and updated any in-text citations to match accordingly.

**Additional Editor Comments:**
This is a thoughtfully conducted and well-articulated realist process evaluation of a co-designed leaflet intervention to improve dementia care information delivery. The theoretical underpinnings, methodological rigor, and stakeholder engagement are major strengths. The manuscript contributes significantly to understanding information provision practices in real-world clinical settings.
However, some aspects warrant further attention and clarification.

**Major Points**

1. **The manuscript is substantially long, especially in the Results and Discussion sections. While the data richness is appreciated, several points are repeated across sections (e.g., distinctions between blanket vs. targeted leaflet distribution). A more concise presentation would improve accessibility. Suggestion: Condense sub-sections by synthesising similar patterns and using summary tables where applicable.**

Thank you for this suggestion. I have revised the Results and Findings sections, streamlining the text by synthesising similar patterns and removing repetition.

2. **The study is highly contextualised in the North-East England setting. The Discussion lacks reflection on how transferable the findings or intervention are to other contexts or systems.
   Suggestion: Include a subsection discussing what aspects of the intervention could be adapted to other settings and what contextual factors are essential for replication.**

Thank you for this comment. The study was conducted within community-based mental health services in a single locality in North-East England, which hosted the co-creation initiative and thus defined the context of the intervention. While the intervention itself may not be directly replicable in other settings, the mechanisms identified through the CMO configurations are potentially transferable. This reflects a key strength of realist evaluation, which focuses on understanding transferable mechanisms rather than interventions as fixed entities. In the revised manuscript I have highlighted this feature of the work.

3. **While the manuscript positions itself clearly as a process evaluation, some outcome data (even preliminary or proxy indicators) would enhance interpretability. For instance, were there any observed increases in service uptake via the leaflet? Suggestion: Consider briefly mentioning if any complementary outcome measures were collected or are planned.**

Thank you for this comment. Quantitative data on service use outcomes, as originally planned in the evaluation protocol, were not collected. Data collection occurred during the COVID-19 pandemic, which disrupted the dissemination of the leaflet in practice and service provision, making it particularly difficult to assess any association between the intervention and changes in service uptake. This limitation is acknowledged in the Discussion and could be addressed in future research.

4. **The point about the leaflet lacking inclusivity for LGBTQ+ individuals and people on service borders is important but underexplored. Suggestion: Expand briefly on how future iterations of the leaflet might better address equity and intersectionality.**

Thank you for this comment. I agree that equity and intersectionality are important considerations for any health and care intervention. The leaflet was designed to strike a balance between being brief and accessible while providing key information, and adding detailed content for specific groups risks diluting its clarity and impact.

Future iterations could nevertheless consider how best to meet the information needs of specific groups, such as the LGBTQ+ community, people with different dementia diagnoses, or young carers. A co-design process with the relevant communities would be essential to identify appropriate approaches, whether through tailored content or dissemination strategies. For example, the leaflet might include a short section signposting readers to specialist sources and resources (e.g., websites of national advocacy or support organisations), while dissemination could be strengthened by working with organisations, venues, or channels most likely to reach these groups.

For individuals living on service borders, the leaflet is unlikely to be an effective solution. The number of people affected is very small, their information needs are diverse, and these are best addressed directly by professionals who can provide tailored guidance.

**While the co-design approach is well described, its challenges, limitations, and power dynamics are under-discussed. Suggestion: Provide more critical reflection on the co-design process (e.g., representation, decision-making authority, tensions if any).**

The co-design process that underpinned the development of the leaflet is reported in detail in the protocol paper linked to the manuscript (De Poli et al., 2020). In response to the reviewer’s comment, I have revised the section “The Intervention” in the article to include considerations of representation and decision-making. While this addition is necessarily concise due to word count constraints, I believe it provides important context for understanding the co-design approach.

**Minor Points**

- **Terminology Consistency: At times, terms like “leaflet,” “intervention,” and “tool” are used interchangeably. Consistent terminology would reduce reader confusion.**

Thank you for identifying these inconsistencies. I have revised the section *“The Intervention”* to provide a clear definition of the intervention and have updated the article to ensure consistent use of terminology throughout.

- **Figure/Table Callouts: Ensure that all figures/tables (especially Figure 1 and 2) are actually embedded and visually referenced in the manuscript.**

Thank you for flagging this. I have revised the manuscript to ensure it meets PLOS ONE's style requirements.

- **Supplementary Material: Clarify in the main text what the supplementary material contains (e.g., StaRI checklist).**

The only supplementary material provided is the StaRI checklist, which is cross-referenced in the final paragraph of the Data Analysis section of the article.

- **Typographical: A few typos and inconsistencies (e.g., missing prepositions, punctuation) throughout—suggest proofreading for polish.**

 Thank you for pointing this out. I have carefully proofread the manuscript and corrected typographical errors and inconsistencies throughout.

**Responses to Reviewer #1**

1. **It would strengthen the manuscript to include the final version of the information leaflet used in the intervention as a supplementary file. Providing this material would enhance transparency, allow readers to better understand the nature of the intervention, and support potential replication or adaptation in other settings.**

Thank you for this suggestion. I agree that transparency and replicability are important, and for this reason the manuscript describes in detail the content, structure, and layout of the leaflet, as well as the processes surrounding its development and use. I have not included the final leaflet as a supplementary file because it contains identifying details of the study site. Making the site identifiable would risk undermining the anonymity of the setting and, in turn, the confidentiality of participants recruited from that site. Given the level of detail already provided in the text, I believe that including a redacted version would not add significant value beyond what is already available to readers, while preserving both site anonymity and participant confidentiality.

2. **The research has qualitative focus, but even minimal quantitative indicators (e.g., how many leaflets were distributed, changes over time, number of clinics involved, patients reached) could enhance understanding of reach and adoption.**

Thank you for this comment. Information on staffing levels and caseloads of the locality teams involved is reported in the protocol paper linked to the manuscript, and I have now added these details in *The Intervention* section of the manuscript to give an indication of the overall scale of the service and staff engaged. Quantitative data on the reach of the intervention (e.g., number of leaflets distributed, patients reached) were not systematically collected, as it would have been practically impossible to track these across locality teams in the absence of a data infrastructure and given variations in local practices and individual practitioner approaches. Moreover, the qualitative data presented in the article show that some individuals received the intervention more than once, meaning that any quantitative indicators would have been affected by double counting and thus would have been of limited reliability or interpretability.

3. **Though some limitations were discussed in the discussion section, it is better to provide critical reflection on methodological limitations (e.g., potential selection bias, representativeness, sample bias, generalisability, and COVID-related constraints).**

Thank you for this comment. In response, I have re-organised the discussion of the study’s strengths and limitations and expanded the limitations section to provide a more detailed reflection on methodological considerations. This includes discussion of potential selection bias, the influence of organisational gatekeeping, challenges arising from group interviews and hierarchical dynamics, staff turnover, and the impact of the COVID-19 pandemic. I have also clarified that, while the findings are situated in their specific context and point in time, they reflect the experiences of participants in an “average” clinical setting and are intended to be transferable across comparable settings and locations rather than generalisable.

**Responses to Reviewer #2**

1. **Clarify the extent to which people with dementia and carers contributed to the leaflet’s design. If their input was limited, acknowledge this as a limitation.**

To address this point, I have revised *The Intervention* section of the manuscript to provide additional background information on the co-design process that underpinned the development of intervention. This is also reported in detail in the protocol paper linked to the manuscript (De Poli et al., 2020).

2. **Provide actionable steps for integrating the leaflet into care pathways (e.g., mandatory training for professionals, standardized updates via the Dementia Strategy Implementation Group).**

Thank you for this suggestion. I have addressed this point in the Discussion where I outline strategies for integrating and sustaining the use of the leaflet in practice.

3. **Acknowledge the single-site focus (North-East England) and homogeneous participant pool (predominantly NHS staff) as potential limitations.**

Thank you for flagging this point, which echoes one of Reviewer 1’s comments. I have substantially revised the paragraph where I discuss the limitations of my work, including methodological aspects. In the manuscript I acknowledge that the setting of the intervention, a community-based mental health service in the North-East of England which hosted the co-creation initiative, and the study sample (NHS staff working in that setting) represented the context of the intervention. Although the intervention itself may not be directly replicable in other settings, the mechanisms that were identified by the realistic evaluation are potentially transferable.

4. **Explicitly state that this is a process evaluation; recommend future work to assess patient/carer outcomes (e.g., satisfaction, service utilization).**

Thank you for this comment, which is addressed in the last paragraph of the Discussion.
