# Supplementary material for: Realist process evaluation of a co-designed intervention to address the unmet information needs of people with dementia and their family carers
Source: PLoS One. 2026 Jun 24;21(6):e0348633. doi: 10.1371/journal.pone.0348633 (PMC13293420; doi:10.1371/journal.pone.0348633)
Supplement: Peer review history

# Peer review: Decision Letter 0

**DOI:** 10.1371/journal.pone.0348633.r001

**Authors:** Sunil Shrestha

28 May 2025

PONE-D-25-13144

Realist process evaluation of a co-designed intervention to address the unmet information needs of people with dementia and their family carers

PLOS ONE

Dear Dr. De Poli,

Thank you for submitting your manuscript to PLOS ONE. After careful consideration, we feel that it has merit but does not fully meet PLOS ONE’s publication criteria as it currently stands. Therefore, we invite you to submit a revised version of the manuscript that addresses the points raised during the review process.

Please submit your revised manuscript by Jul 12 2025 11:59PM. If you will need more time than this to complete your revisions, please reply to this message or contact the journal office at plosone@plos.org. When you're ready to submit your revision, log on to https://www.editorialmanager.com/pone/ and select the 'Submissions Needing Revision' folder to locate your manuscript file.

Please include the following items when submitting your revised manuscript:

- A rebuttal letter that responds to each point raised by the academic editor and reviewer(s). You should upload this letter as a separate file labeled 'Response to Reviewers'.
- A marked-up copy of your manuscript that highlights changes made to the original version. You should upload this as a separate file labeled 'Revised Manuscript with Track Changes'.
- An unmarked version of your revised paper without tracked changes. You should upload this as a separate file labeled 'Manuscript'.

If you would like to make changes to your financial disclosure, please include your updated statement in your cover letter. Guidelines for resubmitting your figure files are available below the reviewer comments at the end of this letter.

If applicable, we recommend that you deposit your laboratory protocols in protocols.io to enhance the reproducibility of your results. Protocols.io assigns your protocol its own identifier (DOI) so that it can be cited independently in the future. For instructions see: https://journals.plos.org/plosone/s/submission-guidelines#loc-laboratory-protocols. Additionally, PLOS ONE offers an option for publishing peer-reviewed Lab Protocol articles, which describe protocols hosted on protocols.io. Read more information on sharing protocols at https://plos.org/protocols?utm_medium=editorial-email&utm_source=authorletters&utm_campaign=protocols.

We look forward to receiving your revised manuscript.

Kind regards,

Sunil Shrestha

Academic Editor

PLOS ONE

**Journal Requirements:**

1. When submitting your revision, we need you to address these additional requirements.

Please ensure that your manuscript meets PLOS ONE's style requirements, including those for file naming. The PLOS ONE style templates can be found at

https://journals.plos.org/plosone/s/file?id=wjVg/PLOSOne_formatting_sample_main_body.pdf and

https://journals.plos.org/plosone/s/file?id=ba62/PLOSOne_formatting_sample_title_authors_affiliations.pdf

2. In the online submission form, you indicated that “The data that support the findings of this study are available from the corresponding author upon reasonable request”.

All PLOS journals now require all data underlying the findings described in their manuscript to be freely available to other researchers, either a. In a public repository, b. Within the manuscript itself, or c. Uploaded as supplementary information.

This policy applies to all data except where public deposition would breach compliance with the protocol approved by your research ethics board. If your data cannot be made publicly available for ethical or legal reasons (e.g., public availability would compromise patient privacy), please explain your reasons on resubmission and your exemption request will be escalated for approval.

3. Please include your full ethics statement in the ‘Methods’ section of your manuscript file. In your statement, please include the full name of the IRB or ethics committee who approved or waived your study, as well as whether or not you obtained informed written or verbal consent. If consent was waived for your study, please include this information in your statement as well.

4. Please include captions for your Supporting Information files at the end of your manuscript, and update any in-text citations to match accordingly. Please see our Supporting Information guidelines for more information: http://journals.plos.org/plosone/s/supporting-information.

**Additional Editor Comments:**

This is a thoughtfully conducted and well-articulated realist process evaluation of a co-designed leaflet intervention to improve dementia care information delivery. The theoretical underpinnings, methodological rigor, and stakeholder engagement are major strengths. The manuscript contributes significantly to understanding information provision practices in real-world clinical settings.

However, some aspects warrant further attention and clarification.

Major Points

1) The manuscript is substantially long, especially in the Results and Discussion sections. While the data richness is appreciated, several points are repeated across sections (e.g., distinctions between blanket vs. targeted leaflet distribution). A more concise presentation would improve accessibility.

Suggestion: Condense sub-sections by synthesising similar patterns and using summary tables where applicable.

2) The study is highly contextualised in the North-East England setting. The Discussion lacks reflection on how transferable the findings or intervention are to other contexts or systems.

Suggestion: Include a subsection discussing what aspects of the intervention could be adapted to other settings and what contextual factors are essential for replication.

3) While the manuscript positions itself clearly as a process evaluation, some outcome data (even preliminary or proxy indicators) would enhance interpretability. For instance, were there any observed increases in service uptake via the leaflet?

Suggestion: Consider briefly mentioning if any complementary outcome measures were collected or are planned.

4) The point about the leaflet lacking inclusivity for LGBTQ+ individuals and people on service borders is important but underexplored.

Suggestion: Expand briefly on how future iterations of the leaflet might better address equity and intersectionality.

5) While the co-design approach is well described, its challenges, limitations, and power dynamics are under-discussed.

Suggestion: Provide more critical reflection on the co-design process (e.g., representation, decision-making authority, tensions if any).

Minor Points

- Terminology Consistency: At times, terms like “leaflet,” “intervention,” and “tool” are used interchangeably. Consistent terminology would reduce reader confusion.

- Figure/Table Callouts: Ensure that all figures/tables (especially Figure 1 and 2) are actually embedded and visually referenced in the manuscript.

- Supplementary Material: Clarify in the main text what the supplementary material contains (e.g., StaRI checklist).

- Typographical: A few typos and inconsistencies (e.g., missing prepositions, punctuation) throughout—suggest proofreading for polish.

[Note: HTML markup is below. Please do not edit.]

Reviewers' comments:

Reviewer's Responses to Questions

**Comments to the Author**

1. Is the manuscript technically sound, and do the data support the conclusions?

The manuscript must describe a technically sound piece of scientific research with data that supports the conclusions. Experiments must have been conducted rigorously, with appropriate controls, replication, and sample sizes. The conclusions must be drawn appropriately based on the data presented.

Reviewer #1: Yes

Reviewer #2: Yes

**********

2. Has the statistical analysis been performed appropriately and rigorously?

Reviewer #1: N/A

Reviewer #2: I Don't Know

**********

3. Have the authors made all data underlying the findings in their manuscript fully available?

The PLOS Data policy requires authors to make all data underlying the findings described in their manuscript fully available without restriction, with rare exception (please refer to the Data Availability Statement in the manuscript PDF file). The data should be provided as part of the manuscript or its supporting information, or deposited to a public repository. For example, in addition to summary statistics, the data points behind means, medians and variance measures should be available. If there are restrictions on publicly sharing data—e.g. participant privacy or use of data from a third party—those must be specified.

Reviewer #1: Yes

Reviewer #2: Yes

**********

4. Is the manuscript presented in an intelligible fashion and written in standard English?

PLOS ONE does not copyedit accepted manuscripts, so the language in submitted articles must be clear, correct, and unambiguous. Any typographical or grammatical errors should be corrected at revision, so please note any specific errors here.

Reviewer #1: Yes

Reviewer #2: Yes

**********

5. Review Comments to the Author

Please use the space provided to explain your answers to the questions above. You may also include additional comments for the author, including concerns about dual publication, research ethics, or publication ethics. (Please upload your review as an attachment if it exceeds 20,000 characters)

**Reviewer #1:**  Thank you for the opportunity to review this valuable work. I would like to congratulate the author for conducting research on process evaluation of a co-designed intervention to address the unmet information needs of people with dementia and their family carers. The objective of this study is to perform realist process evaluation of an intervention co-designed to meet the information needs of people with dementia and their carers, implemented via Community Mental Health Services for Older People (CMHSOP) in the UK. The author has done a commendable job in writing the manuscript.

The research is founded on a good theoretical framework using realist methodology, qualitative data with thematic analysis to provide valuable insights into how information provision works in practice. The strengths of this study are the author has used a Co-designed approach by developing interventions with stakeholders, including professionals and carers, and the use of multiple qualitative data sources (interviews, focus groups) across different time points adds depth and credibility. Moreover, the manuscript adherence to follows the StaRI checklist, which clearly explains the methods, codes, and findings.

However, I would like to suggest that the author perform the following improvements to articulate the findings more effectively.

It would strengthen the manuscript to include the final version of the information leaflet used in the intervention as a supplementary file. Providing this material would enhance transparency, allow readers to better understand the nature of the intervention, and support potential replication or adaptation in other settings.

The research has qualitative focus, but even minimal quantitative indicators (e.g., how many leaflets were distributed, changes over time, number of clinics involved, patients reached) could enhance understanding of reach and adoption.

Though some limitations were discussed in the discussion section, it is better to provide critical reflection on methodological limitations (e.g., potential selection bias, representativeness, sample bias, generalisability, and COVID-related constraints).

**Reviewer #2:**  Few feedback

Clarify the extent to which people with dementia and carers contributed to the leaflet’s design. If their input was limited, acknowledge this as a limitation.

Provide actionable steps for integrating the leaflet into care pathways (e.g., mandatory training for professionals, standardized updates via the Dementia Strategy Implementation Group)

Acknowledge the single-site focus (North-East England) and homogeneous participant pool (predominantly NHS staff) as potential limitations.

Explicitly state that this is a process evaluation; recommend future work to assess patient/carer outcomes (e.g., satisfaction, service utilization).

**********

6. PLOS authors have the option to publish the peer review history of their article (what does this mean?). If published, this will include your full peer review and any attached files.

If you choose “no”, your identity will remain anonymous but your review may still be made public.

**Do you want your identity to be public for this peer review?**  For information about this choice, including consent withdrawal, please see our Privacy Policy.

Reviewer #1: **Yes:**  Rahi Bikram Thapa

Reviewer #2: No

**********

[NOTE: If reviewer comments were submitted as an attachment file, they will be attached to this email and accessible via the submission site. Please log into your account, locate the manuscript record, and check for the action link "View Attachments". If this link does not appear, there are no attachment files.]

While revising your submission, please upload your figure files to the Preflight Analysis and Conversion Engine (PACE) digital diagnostic tool, https://pacev2.apexcovantage.com/. PACE helps ensure that figures meet PLOS requirements. To use PACE, you must first register as a user. Registration is free. Then, login and navigate to the UPLOAD tab, where you will find detailed instructions on how to use the tool. If you encounter any issues or have any questions when using PACE, please email PLOS at figures@plos.org. Please note that Supporting Information files do not need this step.

# Author comment: Author response to Decision Letter 1

**DOI:** 10.1371/journal.pone.0348633.r002

9 Jan 2026

Dear Editor,

Thank you for the opportunity to revise my manuscript Realist process evaluation of a co-designed intervention to address the unmet information needs of people with dementia and their family carers. I have carefully considered your comments, as well as those of the reviewers, and have addressed them in detail in the revised manuscript and in the accompanying response document. I hope that the revisions are satisfactory and address the concerns raised.

Thank you for your time and consideration.

Best regards

Chiara De Poli

Journal Requirements: When submitting your revision, we need you to address these additional requirements.

1. Please ensure that your manuscript meets PLOS ONE's style requirements, including those for file naming. The PLOS ONE style templates can be found at

https://journals.plos.org/plosone/s/file?id=wjVg/PLOSOne_formatting_sample_main_body.pdf and

https://journals.plos.org/plosone/s/file?id=ba62/PLOSOne_formatting_sample_title_authors_affiliations.pdf

I have revised the manuscript to ensure it meets PLOS ONE's style requirements.

2. In the online submission form, you indicated that “The data that support the findings of this study are available from the corresponding author upon reasonable request”. All PLOS journals now require all data underlying the findings described in their manuscript to be freely available to other researchers, either a. In a public repository, b. Within the manuscript itself, or c. Uploaded as supplementary information. This policy applies to all data except where public deposition would breach compliance with the protocol approved by your research ethics board. If your data cannot be made publicly available for ethical or legal reasons (e.g., public availability would compromise patient privacy), please explain your reasons on resubmission and your exemption request will be escalated for approval.

The full dataset supporting this study’s findings cannot be provided within the manuscript due to the volume of data. It also cannot be uploaded as supplementary information or made publicly available, as doing so would require extensive anonymisation that could remove important contextual detail and compromise the meaning and interpretability of the data.

3. Please include your full ethics statement in the ‘Methods’ section of your manuscript file. In your statement, please include the full name of the IRB or ethics committee who approved or waived your study, as well as whether or not you obtained informed written or verbal consent. If consent was waived for your study, please include this information in your statement as well.

I have now included a complete ethics statement in the Methods section of the manuscript. The statement provides the full name of the ethics committee that approved the study, and details of how informed consent was obtained from participants.

4. Please include captions for your Supporting Information files at the end of your manuscript, and update any in-text citations to match accordingly. Please see our Supporting Information guidelines for more information: http://journals.plos.org/plosone/s/supporting-information.

I have included the caption for my Supporting Information files at the end of the manuscript, and updated any in-text citations to match accordingly.

Additional Editor Comments:

This is a thoughtfully conducted and well-articulated realist process evaluation of a co-designed leaflet intervention to improve dementia care information delivery. The theoretical underpinnings, methodological rigor, and stakeholder engagement are major strengths. The manuscript contributes significantly to understanding information provision practices in real-world clinical settings.

However, some aspects warrant further attention and clarification.

Major Points

1. The manuscript is substantially long, especially in the Results and Discussion sections. While the data richness is appreciated, several points are repeated across sections (e.g., distinctions between blanket vs. targeted leaflet distribution). A more concise presentation would improve accessibility. Suggestion: Condense sub-sections by synthesising similar patterns and using summary tables where applicable.

Thank you for this suggestion. I have revised the Results and Findings sections, streamlining the text by synthesising similar patterns and removing repetition.

2. The study is highly contextualised in the North-East England setting. The Discussion lacks reflection on how transferable the findings or intervention are to other contexts or systems.

Suggestion: Include a subsection discussing what aspects of the intervention could be adapted to other settings and what contextual factors are essential for replication.

Thank you for this comment. The study was conducted within community-based mental health services in a single locality in North-East England, which hosted the co-creation initiative and thus defined the context of the intervention. While the intervention itself may not be directly replicable in other settings, the mechanisms identified through the CMO configurations are potentially transferable. This reflects a key strength of realist evaluation, which focuses on understanding transferable mechanisms rather than interventions as fixed entities. In the revised manuscript I have highlighted this feature of the work.

3. While the manuscript positions itself clearly as a process evaluation, some outcome data (even preliminary or proxy indicators) would enhance interpretability. For instance, were there any observed increases in service uptake via the leaflet? Suggestion: Consider briefly mentioning if any complementary outcome measures were collected or are planned.

Thank you for this comment. Quantitative data on service use outcomes, as originally planned in the evaluation protocol, were not collected. Data collection occurred during the COVID-19 pandemic, which disrupted the dissemination of the leaflet in practice and service provision, making it particularly difficult to assess any association between the intervention and changes in service uptake. This limitation is acknowledged in the Discussion and could be addressed in future research.

4. The point about the leaflet lacking inclusivity for LGBTQ+ individuals and people on service borders is important but underexplored. Suggestion: Expand briefly on how future iterations of the leaflet might better address equity and intersectionality.

Thank you for this comment. I agree that equity and intersectionality are important considerations for any health and care intervention. The leaflet was designed to strike a balance between being brief and accessible while providing key information, and adding detailed content for specific groups risks diluting its clarity and impact.

Future iterations could nevertheless consider how best to meet the information needs of specific groups, such as the LGBTQ+ community, people with different dementia diagnoses, or young carers. A co-design process with the relevant communities would be essential to identify appropriate approaches, whether through tailored content or dissemination strategies. For example, the leaflet might include a short section signposting readers to specialist sources and resources (e.g., websites of national advocacy or support organisations), while dissemination could be strengthened by working with organisations, venues, or channels most likely to reach these groups.

For individuals living on service borders, the leaflet is unlikely to be an effective solution. The number of people affected is very small, their information needs are diverse, and these are best addressed directly by professionals who can provide tailored guidance.

While the co-design approach is well described, its challenges, limitations, and power dynamics are under-discussed. Suggestion: Provide more critical reflection on the co-design process (e.g., representation, decision-making authority, tensions if any).

The co-design process that underpinned the development of the leaflet is reported in detail in the protocol paper linked to the manuscript (De Poli et al., 2020). In response to the reviewer’s comment, I have revised the section “The Intervention” in the article to include considerations of representation and decision-making. While this addition is necessarily concise due to word count constraints, I believe it provides important context for understanding the co-design approach.

Minor Points

• Terminology Consistency: At times, terms like “leaflet,” “intervention,” and “tool” are used interchangeably. Consistent terminology would reduce reader confusion.

Thank you for identifying these inconsistencies. I have revised the section “The Intervention” to provide a clear definition of the intervention and have updated the article to ensure consistent use of terminology throughout.

• Figure/Table Callouts: Ensure that all figures/tables (especially Figure 1 and 2) are actually embedded and visually referenced in the manuscript.

Thank you for flagging this. I have revised the manuscript to ensure it meets PLOS ONE's style requirements.

• Supplementary Material: Clarify in the main text what the supplementary material contains (e.g., StaRI checklist).

The only supplementary material provided is the StaRI checklist, which is cross-referenced in the final paragraph of the Data Analysis section of the article.

• Typographical: A few typos and inconsistencies (e.g., missing prepositions, punctuation) throughout—suggest proofreading for polish.

Thank you for pointing this out. I have carefully proofread the manuscript and corrected typographical errors and inconsistencies throughout.

Responses to Reviewer #1

1. It would strengthen the manuscript to include the final version of the information leaflet used in the intervention as a supplementary file. Providing this material would enhance transparency, allow readers to better understand the nature of the intervention, and support potential replication or adaptation in other settings.

Thank you for this suggestion. I agree that transparency and replicability are important, and for this reason the manuscript describes in detail the content, structure, and layout of the leaflet, as well as the processes surrounding its development and use. I have not included the final leaflet as a supplementary file because it contains identifying details of the study site. Making the site identifiable would risk undermining the anonymity of the setting and, in turn, the confidentiality of participants recruited from that site. Given the level of detail already provided in the text, I believe that including a redacted version would not add significant value beyond what is already available to readers, while preserving both site anonymity and participant confidentiality.

2. The research has qualitative focus, but even minimal quantitative indicators (e.g., how many leaflets were distributed, changes over time, number of clinics involved, patients reached) could enhance understanding of reach and adoption.

Thank you for this comment. Information on staffing levels and caseloads of the locality teams involved is reported in the protocol paper linked to the manuscript, and I have now added these details in The Intervention section of the manuscript to give an indication of the overall scale of the service and staff engaged. Quantitative data on the reach of the intervention (e.g., number of leaflets distributed, patients reached) were not systematically collected, as it would have been practically impossible to track these across locality teams in the absence of a data infrastructure and given variations in local practices and individual practitioner approaches. Moreover, the qualitative data presented in the article show that some individuals received the intervention more than once, meaning that any quantitative indicators would have been affected by double counting and thus would have been of limited reliability or interpretability.

3. Though some limitations were discussed in the discussion section, it is better to provide critical reflection on methodological limitations (e.g., potential selection bias, representativeness, sample bias, generalisability, and COVID-related constraints).

Thank you for this comment. In response, I have re-organised the discussion of the study’s strengths and limitations and expanded the limitations section to provide a more detailed reflection on methodological considerations. This includes discussion of potential selection bias, the influence of organisational gatekeeping, challenges arising from group interviews and hierarchical dynamics, staff turnover, and the impact of the COVID-19 pandemic. I have also clarified that, while the findings are situated in their specific context and point in time, they reflect the experiences of participants in an “average” clinical setting and are intended to be transferable across comparable settings and locations rather than generalisable.

Responses to Reviewer #2

1. Clarify the extent to which people with dementia and carers contributed to the leaflet’s design. If their input was limited, acknowledge this as a limitation.

To address this point, I have revised The Intervention section of the manuscript to provide additional background information on the co-design process that underpinned the development of intervention. This is also reported in detail in the protocol paper linked to the manuscript (De Poli et al., 2020).

2. Provide actionable steps for integrating the leaflet into care pathways (e.g., mandatory training for professionals, standardized updates via the Dementia Strategy Implementation Group).

Thank you for this suggestion. I have addressed this point in the Discussion where I outline strategies for integrating and sustaining the use of the leaflet in practice.

3. Acknowledge the single-site focus (North-East England) and homogeneous participant pool (predominantly NHS staff) as potential limitations.

Thank you for flagging this point, which echoes one of Reviewer 1’s comments. I have substantially revised the paragraph where I discuss the limitations of my work, including methodological aspects. In the manuscript I acknowledge that the setting of the intervention, a community-based mental health service in the North-East of England which hosted the co-creation initiative, and the study sample (NHS staff working in that setting) represented the context of the intervention. Although the intervention itself may not be directly replicable in other settings, the mechanisms that were identified by the realistic evaluation are potentially transferable.

4. Explicitly state that this is a process evaluation; recommend future work to assess patient/carer outcomes (e.g., satisfaction, service utilization).

Thank you for this comment, which is addressed in the last paragraph of the Discussion.

Attachment Submitted filename: Responses to reviewers Dec 2025 FINAL.docx

# Peer review: Decision Letter 1

**DOI:** 10.1371/journal.pone.0348633.r003

**Authors:** Kuo-Cherh Huang

19 Apr 2026

Realist process evaluation of a co-designed intervention to address the unmet information needs of people with dementia and their family carers

PONE-D-25-13144R1

Dear Dr. De Poli,

We’re pleased to inform you that your manuscript has been judged scientifically suitable for publication and will be formally accepted for publication once it meets all outstanding technical requirements.

Within one week, you’ll receive an e-mail detailing the required amendments. When these have been addressed, you’ll receive a formal acceptance letter and your manuscript will be scheduled for publication.

An invoice will be generated when your article is formally accepted. Please note, if your institution has a publishing partnership with PLOS and your article meets the relevant criteria, all or part of your publication costs will be covered. Please make sure your user information is up-to-date by logging into Editorial Manager at Editorial Manager® and clicking the ‘Update My Information' link at the top of the page. For questions related to billing, please contact billing support.

If your institution or institutions have a press office, please notify them about your upcoming paper to help maximize its impact. If they’ll be preparing press materials, please inform our press team as soon as possible -- no later than 48 hours after receiving the formal acceptance. Your manuscript will remain under strict press embargo until 2 pm Eastern Time on the date of publication. For more information, please contact onepress@plos.org.

Kind regards,

Kuo-Cherh Huang

Academic Editor

PLOS One

Additional Editor Comments (optional):

Dear Dr. De Poli,

As regards your revised manuscript, first, the Abstract should not exceed 300 words. In addition, the first and second paragraphs in the Introduction section each comprised a single sentence only. Please amend the issues as needed. Thank you.

Kuo-Cherh Huang

Academic Editor

Reviewers' comments:

Reviewer's Responses to Questions

**Comments to the Author**

1. If the authors have adequately addressed your comments raised in a previous round of review and you feel that this manuscript is now acceptable for publication, you may indicate that here to bypass the “Comments to the Author” section, enter your conflict of interest statement in the “Confidential to Editor” section, and submit your "Accept" recommendation.

Reviewer #1: All comments have been addressed

**********

2. Is the manuscript technically sound, and do the data support the conclusions?

The manuscript must describe a technically sound piece of scientific research with data that supports the conclusions. Experiments must have been conducted rigorously, with appropriate controls, replication, and sample sizes. The conclusions must be drawn appropriately based on the data presented.

Reviewer #1: Yes

**********

3. Has the statistical analysis been performed appropriately and rigorously?

Reviewer #1: Yes

**********

4. Have the authors made all data underlying the findings in their manuscript fully available?

The PLOS Data policy requires authors to make all data underlying the findings described in their manuscript fully available without restriction, with rare exception (please refer to the Data Availability Statement in the manuscript PDF file). The data should be provided as part of the manuscript or its supporting information, or deposited to a public repository. For example, in addition to summary statistics, the data points behind means, medians and variance measures should be available. If there are restrictions on publicly sharing data—e.g. participant privacy or use of data from a third party—those must be specified.

Reviewer #1: Yes

**********

5. Is the manuscript presented in an intelligible fashion and written in standard English?

PLOS ONE does not copyedit accepted manuscripts, so the language in submitted articles must be clear, correct, and unambiguous. Any typographical or grammatical errors should be corrected at revision, so please note any specific errors here.

Reviewer #1: Yes

**********

6. Review Comments to the Author

Please use the space provided to explain your answers to the questions above. You may also include additional comments for the author, including concerns about dual publication, research ethics, or publication ethics. (Please upload your review as an attachment if it exceeds 20,000 characters)

Reviewer #1: The author has addressed all the concerns raised and provided reasonable rebuttals where appropriate. I have no further comments.

**********

7. PLOS authors have the option to publish the peer review history of their article (what does this mean?). If published, this will include your full peer review and any attached files.

If you choose “no”, your identity will remain anonymous but your review may still be made public.

**Do you want your identity to be public for this peer review?**  For information about this choice, including consent withdrawal, please see our Privacy Policy.

Reviewer #1: **Yes:**  Rahi Bikram Thapa

**********

# Editor report: Acceptance letter

**DOI:** 10.1371/journal.pone.0348633.r004

**Authors:** Kuo-Cherh Huang

PONE-D-25-13144R1

PLOS One

Dear Dr. De Poli,

I'm pleased to inform you that your manuscript has been deemed suitable for publication in PLOS One. Congratulations! Your manuscript is now being handed over to our production team.

At this stage, our production department will prepare your paper for publication. This includes ensuring the following:

* All references, tables, and figures are properly cited

* All relevant supporting information is included in the manuscript submission,

* There are no issues that prevent the paper from being properly typeset

You will receive further instructions from the production team, including instructions on how to review your proof when it is ready. Please keep in mind that we are working through a large volume of accepted articles, so please give us a few days to review your paper and let you know the next and final steps.

Lastly, if your institution or institutions have a press office, please let them know about your upcoming paper now to help maximize its impact. If they'll be preparing press materials, please inform our press team within the next 48 hours. Your manuscript will remain under strict press embargo until 2 pm Eastern Time on the date of publication. For more information, please contact onepress@plos.org.

You will receive an invoice from PLOS for your publication fee after your manuscript has reached the completed accept phase. If you receive an email requesting payment before acceptance or for any other service, this may be a phishing scheme. Learn how to identify phishing emails and protect your accounts at https://explore.plos.org/phishing.

If we can help with anything else, please email us at customercare@plos.org.

Thank you for submitting your work to PLOS ONE and supporting open access.

Kind regards,

PLOS ONE Editorial Office Staff

on behalf of

Dr. Kuo-Cherh Huang

Academic Editor

PLOS One
